# Editorial: Tolerating Factor VIII: Novel Strategies to Prevent and Reverse Neutralizing Anti-FVIII Antibodies

**DOI:** 10.3389/fimmu.2020.639386

**Published:** 2021-01-25

**Authors:** Sébastien Lacroix-Desmazes, Kathleen P. Pratt

**Affiliations:** ^1^Centre de Recherche des Cordeliers, INSERM, Sorbonne Université, Université de Paris, Paris, France; ^2^Department of Medicine, Uniformed Services University of the Health Sciences, Bethesda, MD, United States

**Keywords:** factor VIII, protein immunogenicity, hemophilia A, immune tolerance induction, anti-drug antibodies

Immunoglobulins are glycoproteins that are present abundantly in blood. They are a major constituent of the immune system and play an important role in protecting the organism from external assaults. This is well illustrated by the fact that immunoglobulins appeared in evolution ~500 million years ago and are present in all vertebrate organisms (1). Immunoglobulins play an essential role in defence against pathogens (2). They are the product of the adaptive branch of the immune system and, more specifically, of B lymphocytes; following complex and iterative cycles of activation, affinity maturation, and selection, B cells have the potential to produce high affinity antibodies with exquisite specificity for their target antigen (2, 3). They also produce promiscuous antibodies (4). Immunoglobulins bridge cellular immune effectors, thus ensuring a coordinated action of the innate and adaptive immune responses (5). Although discussed somewhat less often in the scientific community, immunoglobulins also play an important role in the maintenance of immune homeostasis: participating in the selection of immune cell repertoires, helping to maintain tolerance to Self (6) and building networks of interactions that ensure dynamic physiological immune processes. Nonetheless, under certain conditions, immunoglobulins can turn into foes, for instance, when individuals develop antibodies to innocuous molecules, leading to allergic reactions, or to self-molecules, leading to autoimmune manifestations. Another example is when antibodies neutralize the therapeutic potential of drugs.

Hemophilia A is a rare X-linked bleeding disorder that results from insufficient activity of the pro-coagulant protein factor VIII (FVIII). The administration of exogenous therapeutic FVIII to achieve adequate hemostasis is complicated, in up to 30% of treated patients, by the development of neutralizing anti-FVIII IgG, referred to as “FVIII inhibitors” (7). The occurrence of intractable FVIII inhibitors has been associated with increased patient morbidity and mortality (8). Until recently, the only clinical options to tackle the problem of FVIII inhibitor development were intensive FVIII treatment, termed “Immune Tolerance Induction (ITI)” (9), or use of recombinant activated factor VII or a pro-coagulant protein cocktail such as Factor Eight Inhibitor Bypass Agent (FEIBA) to achieve hemostasis without relying on FVIII (10–12). Although ITI succeeds in eliminating or greatly reducing inhibitor titers in 60%–80% of treated patients, both ITI and repeated administrations of these ‘bypass’ agents are extremely costly, thereby presenting a clear societal burden and making them unavailable to many inhibitor patients worldwide (13). Furthermore, ITI and bypass treatment regimens are extremely cumbersome for the patients, their families and clinicians. Although inhibitor eradication is more readily achieved in inhibitor patients with low initial titers (“low responders”) (14), the clinical/scientific rationale allowing one to confidently anticipate success or failure of ITI in patients with higher-titer inhibitors is still incomplete. Clearly, new approaches are required to understand and reduce the immunogenicity of therapeutic FVIII and to impart tolerance to FVIII or facilitate elimination of FVIII inhibitors when they have developed.

This Research Topic focuses on promising recent approaches to promote durable immune tolerance to FVIII, whether administered exogenously or through gene therapy. That said, we acknowledge that these tolerogenic therapies must henceforth be evaluated in the context of impressive advances in development of various new bypass therapies as alternatives to FVIII replacement therapy, several of which are already in the clinic. These bypass agents may be administered as a FVIII alternative, or in some cases together with FVIII in the presence or absence of immunomodulatory agents. For example, in 2012, a groundbreaking report described a bispecific monoclonal antibody that crosslinks factor IXa and its substrate, factor X, with the appropriate spacing and orientation as well as affinity to mimic FVIII cofactor activity, and that corrected clotting in a nonhuman primate model in the presence of neutralizing anti-FVIII antibodies (15). Less than 10 years later, the latest generation of this therapeutic, Emicizumab, has radically transformed the landscape of hemophilia care: most notably by providing a long-lived hemostatic agent for inhibitor patients, but also as a potential alternative to FVIII for non-inhibitor patients. It is fascinating to realize that a coagulation disorder caused by neutralizing antibodies could be corrected (although not in every clinical scenario) through use of a rationally engineered therapeutic antibody! In addition to established, although imperfect, agents for inhibitor management, such as activated factor VII and procoagulant protein cocktails, molecules that block anti-thrombotic feedback loops in the coagulation cascade are being evaluated as potential bypass agents (16). By-passing agents are, however, not as efficient as FVIII in situations of major bleeding or surgery, and they may carry potentially life-threatening pro-thrombotic potency in some patients and in certain clinical situations (17). Our position, at this point in time, is that tolerizing patients to FVIII will remain a central goal of hemophilia A therapy as long as patients continue to receive FVIII replacement therapy, and as long as patients choosing alternative therapies experience breakthrough bleeds and undergo surgeries that necessitate administering FVIII in the absence of inhibitory antibodies.

With ten general review articles and nine original research articles, the present Research Topic entitled “Tolerating Factor VIII: Novel Strategies to Prevent and Reverse Anti-FVIII Inhibitors” presents some of the latest advances in our understanding of FVIII immunogenicity in hemophilia A patients and describes promising strategies to control anti-FVIII immune responses.

Review articles by Lacroix-Desmazes et al. and by Merlin and Follenzi together present a broad overview of FVIII immunogenicity and describe novel approaches to reduce FVIII immunogenicity and induce tolerance to FVIII, most of which are still in the basic science/preclinical evaluation stage. Abdi et al. present a systematic review and meta-analysis to estimate the prevalence and incidence of *non-neutralizing* anti-FVIII antibodies in hemophilia A patients (which are often not measured clinically but are clearly relevant to FVIII immunogenicity). Peyvandi et al. review possible FVIII product-related differences that could affect its immunogenicity and discuss potential factors contributing to the lower apparent immunogenicity of plasma-derived FVIII, compared to recombinant FVIII, that was seen in the prospective, randomized SIPPET clinical trial. Hart elegantly describes *in vitro*, *in silico* and epidemiological methods to predict inhibitor risk in non-severe hemophilia A, which is caused by dysfunctional rather than missing FVIII and therefore presents the opportunity to evaluate individual disease-causing mutations and their associated effects on binding MHC Class II. Several additional reviews explore potential interventions to promote immune tolerance to FVIII. Mimoun et al. review the role of FcRn-mediated cross-placental transfer of IgGs in promoting tolerance, and the potential of exploiting this process through administering recombinant Fc-fusion proteins such as FVIII-Fc. FVIII immunogenicity in preclinical models of gene therapy and in recent clinical trials is addressed in reviews by Patel et al. and Samelson-Jones and Arruda, while the potential use of platelet-targeted FVIII gene therapy to restore hemostasis, even in the presence of inhibitory antibodies, is reviewed by Cai and Shi. The concern of potential inhibitor development in patients treated with FVIII gene therapy is addressed by original research from Biswas et al., in which mice that developed inhibitors following AAV-based gene therapy showed improvement when B-cell depletion was combined with rapamycin.

The importance of inflammatory processes and roles of immunoregulatory enzymes such as heme oxygenase-1 and Indoleamine 2,3 dioxygenase in promoting hemophilic inhibitor responses versus tolerance to administered FVIII are reviewed by Matino et al.; this review sets the stage nicely for the original research article by Karim et al. in which RNASeq/transcriptomics analysis of peripheral blood mononuclear cells isolated from inhibitor subjects and controls identified up-regulated genes implicating specific inflammatory and innate immune processes in the maintenance of FVIII inhibitors. Regarding product-related differences, Zakas et al. report that partial oxidation of a recombinant FVIII product does not affect its tendency to aggregate, suggesting that the observed heightened immunogenicity of oxidized FVIII (in an animal model) was likely not due to aggregation-induced immune complex formation. Deliberate modification of recombinant FVIII to influence its immunogenicity is described by Delignat et al., where they demonstrate the importance of mannose-ending glycans on FVIII for its immune recognition, and by Georgescu et al. reporting inhibition of B-cell activation by a recombinant FVIII-Fc protein.

Animal model studies evaluating additional novel interventions besides FVIII protein modification include enlistment of engineered, FVIII-specific T-regulatory cells (De Paula Pohl et al.) and a recombinant murine Fc-IL-2 fusion protein that expands T-regulatory cells (Chen et al.). The potential of oral tolerance achieved *via* delivery of encapsulated FVIII, and mechanisms at play at the level of the intestine, are addressed in original research from Kumar et al. The involvement of Fc gamma receptors and of complement C3 in the development of FVIII inhibitors in preclinical models of hemophilia A are explored in original research from Zerra et al.

Finally, many of the concepts and approaches developed to address hemophilic immune responses may be generalized to other fields wherein neutralizing antibodies and adverse immune responses are a major concern. The case of FVIII inhibitor development is rather unusual, in that development of these anti-drug antibodies does not preclude further treatment with FVIII, including via ITI. This presents us with the opportunity to carry out longitudinal studies of human as well as animal model immune responses to discern immunogenic and tolerogenic mechanisms. We hope that readers of Frontiers in Immunology with expertise in other types of anti-drug antibodies, or in antibody-mediated graft rejection following transplantation, etc., will also find this collection of interest, while it provides a timely and informative snapshot of the field for the hemophilia research community.

## Author Contributions

Both authors wrote the editorial together. All authors contributed to the article and approved the submitted version.

## Funding

Publication charges for this collection were subsidized by an unrestricted, investigator-initiated educational grant from Grifols, Inc. to KP. KP is also funded by NIH R01 HL 130448, R01 HL 126727B, and IAAA-A-HL-007.001 to the Collaborative Health Sciences Research Program of the Uniformed Services University of the Health Sciences. Grifols was not involved in the study design, collection, analysis, interpretation of data, the writing of this article or the decision to submit it for publication. SL-D is supported by INSERM, Centre National de la Recherche Scientifique and Sorbonne Université (Paris, France) and by grants from Agence Nationale de la Recherche (ANR-10-BLAN-1118 and ANR-18-CE17-0010) and from the European Community (H2020-MSCA-ITN-2019 project 859974 EDUC8).

## Disclaimer

The opinions or assertions contained herein are the private ones of the authors and are not to be construed as official or reflecting the views of the Department of Defense or the Uniformed Services University of the Health Sciences.

## Conflict of Interest

KP is an inventor on FVIII patents.

The remaining author declares that the research was conducted in the absence of any commercial or financial relationships that could be construed as a potential conflict of interest.

## References

[1] Flajnik MF A cold-blooded view of adaptive immunity. Nat Rev Immunol (2018) 18:438–53. 10.1038/s41577-018-0003-9 PMC6084782 29556016

[2] Lu LL Suscovich TJ Fortune SM Alter G Beyond binding: antibody effector functions in infectious diseases. Nat Rev Immunol (2018) 18:46–61. 10.1038/nri.2017.106 29063907 PMC6369690

[3] Schroeder HW Jr. Cavacini L Structure and function of immunoglobulins. J Allergy Clin Immunol (2010) 125:S41–52. 10.1016/j.jaci.2009.09.046 PMC3670108 20176268

[4] Dimitrov JD Planchais C Roumenina LT Vassilev TL Kaveri SV Lacroix-Desmazes S Antibody polyreactivity in health and disease: statu variabilis. J Immunol (2013) 191:993–9. 10.4049/jimmunol.1300880 23873158

[5] Bournazos S Wang TT Dahan R Maamary J Ravetch JV Signaling by Antibodies: Recent Progress. Annu Rev Immunol (2017) 35:285–311. 10.1146/annurev-immunol-051116-052433 28446061 PMC5613280

[6] Heyman B Regulation of antibody responses *via* antibodies, complement, and Fc receptors. Annu Rev Immunol (2000) 18:709–37. 10.1146/annurev.immunol.18.1.709 10837073

[7] Ehrenforth S Kreuz W Scharrer I Linde R Funk M Gungor T Incidence of development of factor VIII and factor IX inhibitors in haemophiliacs. Lancet (1992) 339:594–8. 10.1016/0140-6736(92)90874-3 1347102

[8] Walsh CE Jimenez-Yuste V Auerswald G Grancha S The burden of inhibitors in haemophilia patients. Thromb Haemost (2016) 116 Suppl 1:S10–7. 10.1160/TH16-01-0049 27528280

[9] Hay CR Dimichele DM International Immune Tolerance, S The principal results of the International Immune Tolerance Study: a randomized dose comparison. Blood (2012) 119:1335–44. 10.1182/blood-2011-08-369132 22101900

[10] Rocino A Franchini M Coppola A Treatment and Prevention of Bleeds in Haemophilia Patients with Inhibitors to Factor VIII/IX. J Clin Med (2017) 6:11–8. 10.3390/jcm6040046 PMC5406778 28420167

[11] Astermark J Donfield SM Dimichele DM Gringeri A Gilbert SA Waters J A randomized comparison of bypassing agents in hemophilia complicated by an inhibitor: the FEIBA NovoSeven Comparative (FENOC) Study. Blood (2007) 109:546–51. 10.1182/blood-2006-04-017988 16990605

[12] Leissinger C Gringeri A Antmen B Berntorp E Biasoli C Carpenter S Anti-inhibitor coagulant complex prophylaxis in hemophilia with inhibitors. N Engl J Med (2011) 365:1684–92. 10.1056/NEJMoa1104435 22047559

[13] D’angiolella LS Cortesi PA Rocino A Coppola A Hassan HJ Giampaolo A The socioeconomic burden of patients affected by hemophilia with inhibitors. Eur J Haematol (2018) 101:435–56. 10.1111/ejh.13108 29889317

[14] Van Den Berg HM Mancuso ME Konigs C D’oiron R Platokouki H Mikkelsen TS ITI Treatment is not First-Choice Treatment in Children with Hemophilia A and Low-Responding Inhibitors: Evidence from a PedNet Study. Thromb Haemost (2020) 120:1166–72. 10.1055/s-0040-1713097 32572865

[15] Kitazawa T Igawa T Sampei Z Muto A Kojima T Soeda T A bispecific antibody to factors IXa and X restores factor VIII hemostatic activity in a hemophilia A model. Nat Med (2012) 18:1570–4. 10.1038/nm.2942 23023498

[16] Weyand AC Pipe SW New therapies for hemophilia. Blood (2019) 133:389–98. 10.1182/blood-2018-08-872291 30559264

[17] Aledort L Mannucci PM Schramm W Tarantino M Factor VIII replacement is still the standard of care in haemophilia A. Blood Transfus (2019) 17:479–86. 10.2450/2019.0211-19 PMC6917528 31846611

